# The effects of context processing on social cognition impairments in adults with Asperger's syndrome

**DOI:** 10.3389/fnins.2014.00270

**Published:** 2014-09-03

**Authors:** Sandra Baez, Agustin Ibanez

**Affiliations:** ^1^Institute of Cognitive Neurology (INECO) and Institute of Neuroscience, Favaloro UniversityBuenos Aires, Argentina; ^2^UDP-INECO Foundation Core on Neuroscience (UIFCoN), Diego Portales UniversitySantiago, Chile; ^3^National Scientific and Technical Research Council (CONICET)Buenos Aires, Argentina; ^4^Universidad Autónoma del CaribeBarranquilla, Colombia; ^5^Australian Research Council, Centre of Excellence in Cognition and its Disorders SydneyNSW, Australia

**Keywords:** autism spectrum disorders, Asperger's syndrome, social cognition, social context processing, contextual cues

## Abstract

Social cognition—the basis of all communicative and otherwise interpersonal relationships—is embedded in specific contextual circumstances which shape intrinsic meanings. This domain is compromised in the autism spectrum disorders (ASDs), including Asperger's syndrome (AS) (DSM-V). However, the few available reports of social cognition skills in adults with AS have largely neglected the effects of contextual factors. Moreover, previous studies on this population have also failed to simultaneously (a) assess multiple social cognition domains, (b) examine executive functions, (c) follow strict sample selection criteria, and (d) acknowledge the cognitive heterogeneity typical of the disorder. The study presently reviewed (Baez et al., 2012), addressed all these aspects in order to establish the basis of social cognition deficits in adult AS patients. Specifically, we assessed the performance of AS adults in multiple social cognition tasks with different context-processing requirements. The results suggest that social cognition deficits in AS imply a reduced ability to implicitly encode and integrate contextual cues needed to access social meaning. Nevertheless, the patients' performance was normal when explicit social information was presented or when the situation could be navigated with abstract rules. Here, we review the results of our study and other relevant data, and discuss their implications for the diagnosis and treatment of AS and other neuropsychiatric conditions (e.g., schizophrenia, bipolar disorder, frontotemporal dementia). Finally, we analyze previous results in the light of a current neurocognitive model of social-context processing.

**Social cognition** processes are embedded in specific contextual circumstances which shape intrinsic social meaning (Ibañez and Manes, 2012; Kennedy and Adolphs, 2012). The notion of social cognition involves several domains, such as emotion recognition, theory of mind (ToM), decision-making, empathy, moral judgment, knowledge of social norms, among others. Despite their differences, some of these domains involve similar underlying processes. These include spontaneous perception and interpretation of relevant situational elements to construct a given social context (Klin, 2000) through implicit inference of **contextual cues** which bias an action's social meaning (Ibañez and Manes, 2012). For example, facial emotion recognition is a context-sensitive process. Real-life facial expressions are typically embedded in a rich, informative context. Visual scenes, voices, bodies, other faces, and even words influence how an emotion is perceived in a face (Barrett et al., 2011). In contrast, other social cognition processes involve the use of explicit and abstract rules about the conventions or expected behaviors framing a social setting (e.g., explicit social norms during specific social interactions). In brief, different social cognition domains may involve different strategies.

KEY CONCEPT 1. Social cognition A complex set of processes subserving adaptive social interactions.

KEY CONCEPT 2. Contextual cues Intrinsic aspects of the cognitive processes that enable understanding of an object or stimulus.

This view aligns with the second-person approach to social cognition, which suggests that interpersonal understanding is primarily a matter of social interaction and emotional engagement with others. Thus, cognitively speaking, there are fundamental differences between interacting with others and merely observing them (Schilbach, 2010, 2014; Schilbach et al., 2013). In support of this approach, a functional magnetic resonance imaging study with healthy participants showed that social context significantly changes the neural underpinnings of action control (Schilbach et al., 2011). This finding demonstrates that performing incongruent actions in the presence of a virtual other (as compared with non-social cues) differentially increases neural activity in regions supporting action monitoring, response inhibition, and social cognition.

Most neuropsychiatric conditions are characterized by social cognition deficits and/or abnormal activation of social brain areas (Kennedy and Adolphs, 2012; Millan et al., 2012; Ibanez et al., 2014). Indeed, psychiatric disorders may be conceptualized as disorders of implicit social interaction, rather than impairments of explicit social cognition (Schilbach et al., 2013). For instance, difficulty in social functioning is a key diagnostic criterion for several psychiatric disorders (Kennedy and Adolphs, 2012), such as autism spectrum disorders (ASDs). Asperger's syndrome (AS) is a variant of this spectrum. It is characterized by severe, sustained impairments in social interaction and restricted, repetitive patterns of behavior, interest, and activities (American Psychiatric Association, 1994). AS has been removed from the DSM-V as an explicit diagnostic category. However, individuals previously diagnosed with it still require specific assessment and treatment given their impairments in occupational and otherwise social settings.

This review addresses social cognition in adults with AS, focusing on a recent study (Baez et al., 2012) reporting deficits in social/contextual processing. In addition, our analysis covers other studies which offer insights into the diagnosis and treatment of AS and other neuropsychiatric disorders. Finally, the available evidence is analyzed in terms of a current neurocognitive model of social context processing.

## Social cognition deficits in adults with asperger's syndrome

Contextual effects are present at every level, from basic perception to social interaction. Contextual sensitivity guides our perception. It helps us to focus on relevant social cues, ignore irrelevant details, and understand incomplete or ambiguous information. In adults with AS, impaired social cognition abilities, such as emotion recognition, ToM, empathy, and moral judgment, are related to contextual sensitivity (Vermeulen, 2012). Nonetheless, contextual effects on social cognition performance in AS are not well-understood.

Individuals with AS exhibit deficits in **emotion recognition** from faces (Philip et al., 2010), particularly those conveying negative emotions (Ashwin et al., 2007; Falkmer et al., 2011; Doi et al., 2013). Also, studies using the Faux Pas Test (FPT) have revealed **ToM** impairments, especially in understanding the intentions (cognitive ToM) and the emotional impact of others' actions (affective ToM) (Zalla et al., 2009; Gonzalez-Gadea et al., 2013). However, evidence obtained through the Reading-the-Mind-in-the-Eyes Test (RMET) has been inconsistent, with mixed reports of impaired (Baron-Cohen et al., 1997, 2001) and preserved (Roeyers et al., 2001; Ponnet et al., 2004; Spek et al., 2010; Gonzalez-Gadea et al., 2013; Lugnegard et al., 2013) performance. These controversial results may be explained by cultural factors (Roeyers et al., 2001; Spek et al., 2010) or by design features of the RMET, since it only weakly correlates with other ToM measures (Luzzatti et al., 2002; Spek et al., 2010).

KEY CONCEPT 3. Emotion recognition Ability to recognize affective states in another person.

KEY CONCEPT 4. ToM Ability to infer the beliefs, intentions, and emotions of others.

Other studies on AS have revealed impairments in cognitive and affective **empathy** through self-report questionnaires (Baron-Cohen and Wheelwright, 2004; Rogers et al., 2007) and experimental designs (Dziobek et al., 2008). Finally, as regards **moral judgment**, adults with AS exhibit decreased levels of emotional reaction to moral dilemmas (Gleichgerrcht et al., 2013) and atypical moral judgments when they need to consider both the intention to harm (accidental vs. intentional) and the outcome (neutral vs. negative) of a person's actions (Moran et al., 2011).

KEY CONCEPT 5. Empathy Capacity to share and understand the emotional states of others by reference to oneself.

KEY CONCEPT 6. Moral judgment Moral reasoning process required to define whether an action is morally right or wrong.

## Relevant factors in the assessment of social cognition in asperger's syndrome

Despite the evidence above, previous studies have neglected crucial factors to establish the basis of social functioning impairments in this population. First, the exploration of social cognition deficits in AS requires tapping multiple domains with implicit and explicit tasks. Implicit social cognition tasks require the spontaneous perception and interpretation of relevant situational elements to construct a given social context. For example, when we see a person in physical pain (e.g., being stepped on by someone), our appraisal of the context determines our empathic response. We would feel more empathy if the pain results from a deliberate attack than if it is caused by accident. Conversely, in explicit social cognition tasks, situational elements are clearly defined and can usually be analyzed with reference to universal, explicitly learned rules. For instance, the identification of social misbehavior (e.g., touching a stranger on the street) depends on explicit norms that we learn through experience.

Recent relevant studies have over-emphasized explicit forms of social cognition in adults with AS (Schilbach et al., 2013). However, the evidence (Klin, 2000; Senju et al., 2009; Izuma et al., 2011; Baez et al., 2012; Schilbach et al., 2012) suggests that explicit social cognition processes are not impaired in this population. Instead, deficits emerge in implicit processes that contribute to social interaction and allow the automatic integration of relevant social cues in more complex situations (Schilbach et al., 2012). For instance, Senju et al. (2009) found that the eye movements of AS individuals (as opposed to those of neurotypical adults) do not anticipate others' actions in a non-verbal false belief task. Thus, these individuals do not attribute mental states spontaneously, although they may be able to do so in explicit tasks.

In a more recent study, Schilbach et al. (2012) used a stimulus-response compatibility paradigm to investigate the effect of social gaze on action control in high functioning autism (HFA) individuals. When control participants were being looked at by a virtual other, they took significantly less time to generate a spatially incongruent response. This effect was not present in adults with HFA. According to the authors, the effect observed in healthy participants suggests that social cues trigger motor preparatory programs that may help to coordinate one's actions with those of someone else.

In sum, the evidence suggests that individuals with AS cannot spontaneously apply social reasoning abilities to solve more naturalistic tasks; however, their performance improves when explicit information is provided (Klin, 2000; Izuma et al., 2011), therefore the use of both implicit and explicit tasks affords a more comprehensive evaluation which may reveal whether the varied social cognition deficits observed in AS are related to a common underlying factor.

Second, most previous reports of social cognition deficits in AS (Baron-Cohen et al., 2001; Baron-Cohen and Wheelwright, 2004; Moran et al., 2011; Zalla et al., 2011) also included patients with other ASDs (e.g., HFA). The differentiation among autistic subtypes, especially between AS and HFA, is still matter of debate. Still, the evidence suggests that these disorders should be studied as separate diagnostic entities (for a review see Matson and Wilkins, 2008). For instance, unlike HFA, AS does not involve delays in early cognitive functioning (Frith, 2004). Relative to HFA individuals, adults with AS have greater visual-motor deficits (Klin et al., 1995; Noterdaeme et al., 2010), less strong impairments in verbal comprehension (Noterdaeme et al., 2010; Planche and Lemonnier, 2012), higher verbal than performance IQ (Klin et al., 1995), and less severe behavioral abnormalities (Gilchrist et al., 2001). Therefore, these cognitive and behavioral differences may bias the results of social cognition studies.

Third, EFs are required for processing emotional stimuli and social cognition tasks (Pessoa, 2008; Uekermann et al., 2010). During emotional processing, stimuli must be held in working memory while and irrelevant information is inhibited. Similarly, ToM and empathy entail working memory storage and switching between one's own perspective and that of another person (Uekermann et al., 2010). Nevertheless, no studies with AS patients have controlled for the effect of EF on social cognition performance. Finally, it is important to consider that adult AS groups exhibit great inter-subject variability in multiple domains (Hill and Bird, 2006; Towgood et al., 2009), as shown by EF and social cognition tasks. This means that the AS population includes patients with both sub-normal and supra-normal performance.

The study targeted in the present review (Baez et al., 2012) addressed all these factors. Its main aim was to assess multiple social cognition domains through tasks with different levels of contextual dependence, while assessing the influence of EF. In addition, it explored inter-individual variability among AS patients. This was done through multiple case series analysis (Hill and Bird, 2006; Towgood et al., 2009), a methodology to detect the domains in which a given individual displays abnormal performance. The sample comprised 30 participants: 15 adults who met the DSM-IV criteria for AS (American Psychiatric Association, 1994) and 15 healthy controls. The social cognition domains evaluated were emotion recognition, ToM, empathy, moral judgment, knowledge of social norms, and self-monitoring behavior in social settings.

We included tasks with different levels of contextual dependence and involvement of real-life scenarios: (a) emotion recognition was assessed through the Awareness of Social Inference Test (TASIT) (McDonald et al., 2003, 2006; Kipps et al., 2009), a task with high context-processing requirements; (b) emotional and cognitive aspects of ToM were evaluated with the RMET (Baron-Cohen et al., 1997) and the FPT (Stone et al., 1998); (c) the cognitive and affective components of empathy were examined through an ecological Empathy for Pain Task (EPT) (Decety et al., 2012; Baez et al., 2013a) and the Interpersonal Reactivity Index (Davis, 1983); (d) the contributions of intentions and outcomes to moral judgment were explored with a well-characterized moral task (Young et al., 2010; Baez et al., 2014a); (e) self-monitoring skills were assessed using the Revised Self-Monitoring Scale (Lennox and Wolfe, 1984); and (f) knowledge of social norms was evaluated through an explicit (abstract and context-independent) instrument, namely, the Social Norms Questionnaire (SNQ).

## Deficits in contextual social cognition

**Figure 1:**
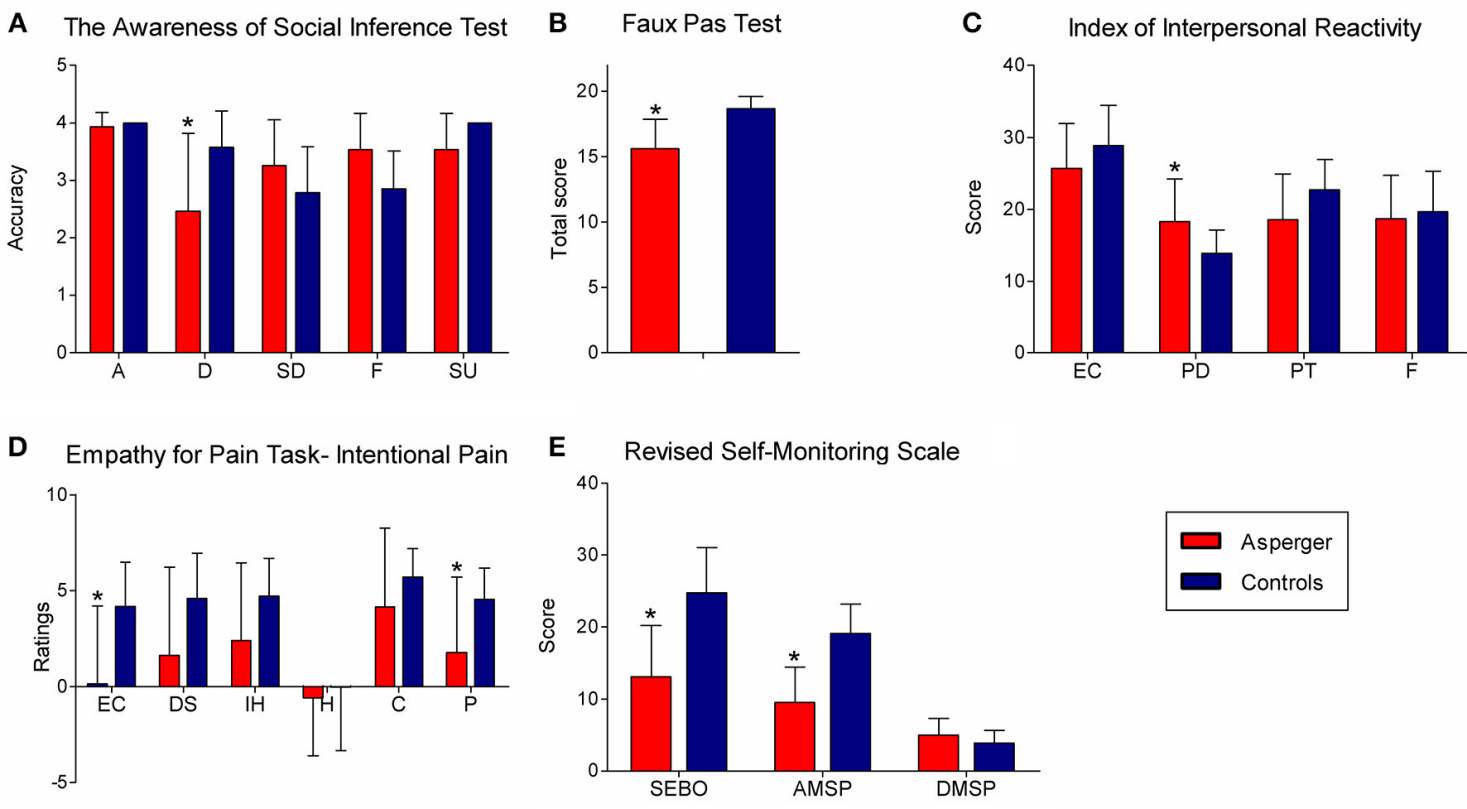
**Significant differences between AS adults and controls in social cognition tasks (Baez et al., 2012). (A)** TASIT (accuracy per category). A, anger; D, disgust; SD, sadness; F, fear; SU, surprise. **(B)** Faux Pas Test (total score). **(C)** Scores on IRI subscales. EC, empathic concern; PD, personal distress; PT, perspective taking; F, fantasy. **(D)** Empathy-for-Pain Task (ratings for intentional pain situations). EC, empathic concern; DS, discomfort; IH, intention to hurt; H, happiness; C, correctness; P, punishment. **(E)** Scores on RSMS subscales. SEBO, sensitivity for expression behavior of others; AMSP, ability to modify self-presentation; DMSP, difficulty to modify self-presentation. Asterisks indicate significant differences.

**Figure 2:**
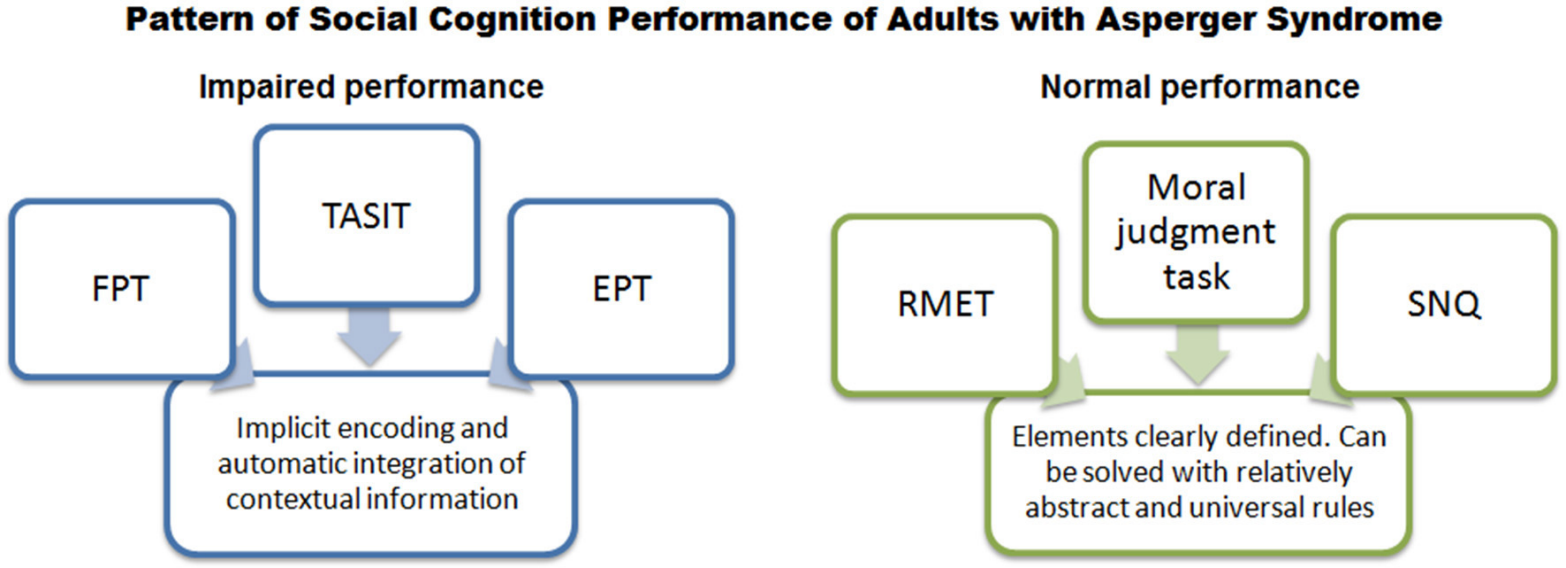
**Pattern of performance of adults with AS in social cognition tasks**. Adults with AS were impaired in tasks that involved implicit encoding and automatic integration of contextual cues to interpret a given social situation. Conversely, they performed as well as controls in tasks which featured well defined-situational elements and could be solved by using relatively abstract, universal rules. FPT, Faux Pas Test; TASIT, The Awareness of Social Inference Test; EPT, Empathy for Pain Task; RMET, Reading the Mind in the Eyes Test; SNQ, Social Norms Questionnaire.

In our study, adults with AS exhibited deficits in multiple social cognition domains (emotion recognition, ToM, empathy, and self-monitoring in social settings) (Figure 1). Specifically, the patients performed poorly on social cognition tasks (TASIT, FPT, EPT) that involve implicit encoding of socially relevant information and automatic integration of contextual information to interpret a given social situation. Conversely, they performed as well as controls in the RMET, the moral judgment task, and the SNQ. These tasks feature clearly defined situational elements and can be solved with relatively abstract universal rules. In coherence with a recent study (Schneider et al., 2013), this pattern of performance suggests that social cognition deficits in the AS population may reflect a single underlying factor: deficits to implicitly encode and integrate contextual information required to construct social meanings (see Figure 2).

Furthermore, our study was the first to explore the effect of EFs on social cognition performance in this population. Adults with AS and controls were similar regarding executive functioning. Moreover, to control for the effect of EF on performance during social cognition tasks, we conducted covariance analyses adjusted for cognitive flexibility—the only domain revealing significant differences. All differences in social cognition measures remained significant. Moreover, we found no significant correlations between the EF measures with higher variability and the social cognition tasks that were different between groups. Taken together, these results indicate that EFs do not play a major role in the observed social cognition impairments.

This study also offered the first analysis of intra-individual variability of social cognition measures in adults with AS. Individual patient analyses revealed sub-normal performance on the same tasks yielding between-group differences. Moreover, their social cognition performance did not follow the same pattern of strengths and weaknesses reported in other cognitive domains (Hill and Bird, 2006; Towgood et al., 2009; Gonzalez-Gadea et al., 2013). Rather, the social cognition patterns of individuals with AS were characterized by sub-normal performance only.

Overall, our results indicate that adults with AS may use abstract rules to compensate their impairments in social cognition. This population has been reported to possess superior abstract reasoning abilities (Hayashi et al., 2008; Soulieres et al., 2011). Such a strength may be beneficial for social cognition tasks that require using abstract rules and integrating explicit information, without improving performance in situations requiring implicit integration of contextual cues. In line with this interpretation, Kuzmanovic et al. (2011) investigated the differential impact of verbal and nonverbal information on interpersonal impression formation in adults with HFA. While both verbal and nonverbal social stimuli had a considerable influence on healthy individuals, the HFA participants tended to rely on the explicit verbal domain, thereby neglecting non-verbal cues. Thus, individuals with HFA seem apply a more analytic and rule-based processing style when dealing with social information. However, in most real-life situations, social demands are not explicitly formulated. The meaning of social information is only partially predictable and relies heavily on context, which reduces the possibility of inference through explicit abstract rules. Instead, social demands must be implicitly inferred by integrating contextual cues. Thus, the pattern of deficits observed in adults with AS may partially explain their daily social interaction difficulties.

Notwithstanding, a recent study showed an opposite pattern of performance. Schwarzkopf et al. (2014) found that visuospatial perspective-taking appears to be intact in HFA participants, although this ability is impaired when used explicitly. The discrepancy between this finding and previous evidence (Klin, 2000; Senju et al., 2009; Izuma et al., 2011; Baez et al., 2012; Schilbach et al., 2012) probably reflects differences between the domains assessed. Schwarzkopf et al. (2014) examined “level 1” visuospatial perspective-taking, that is, the ability to adequately establish what the target person can and cannot see. This skill, however, does not require emotional processing or the inference of others' mental states. Thus, the cognitive processes underlying “level 1” perspective-taking may be different from those involved in social cognition. Both our results and previous evidence suggest a dissociation between impaired implicit and relatively intact explicit levels of social cognition in AS. However, further research is needed to determine which low- and high-level cognitive domains follow this pattern of performance.

## Implications and future directions

Our findings have important clinical implications. Since adequate social responses are crucial for daily functioning, social cognition impairments should be considered in the assessment and treatment of AS individuals. In line with previous suggestions (Klin, 2000; Vermeulen, 2012; Hanley et al., 2013), our study indicates that social cognition deficits in AS are better detected using context-sensitive tasks involving real-life scenarios, as shown in other neuropsychiatric populations (Torralva et al., 2009; Ibañez and Manes, 2012; Baez et al., 2013b, 2014b; Melloni et al., 2014). Such instruments should be used in clinical assessments and empirical research on adults with AS.

Traditional social-skill interventions for individuals with AS are based on learning explicit rules to build and foster relationships (Cappadocia and Weiss, 2011). However, such programs lack ecological validity: the patients fail to generalize their new skills to situations outside the treatment setting (Rao et al., 2008; Cappadocia and Weiss, 2011). These limitations may be circumvented by incorporating naturalistic environments and social context to the intervention materials. Social-skill programs for AS patients should promote the acquisition of implicit rules to navigate unpredictable social contexts. Instead of emphasizing explicit social knowledge, clinicians should focus on promoting contextual sensitivity to different situational configurations (Vermeulen, 2014). This approach may help individuals with AS to better understand the world around them and react more appropriately (Vermeulen, 2012).

Incidentally, note that AS has been excluded as a specific diagnostic category in the DSM-V. Notwithstanding, our findings are still relevant for studying individual differences within ASDs and the subset of people showing the particular profile previously diagnosed as AS. Future studies including comprehensive assessments of cognitive and social domains with larger AS samples may help to identify subcategories in the ASDs.

**Figure 3:**
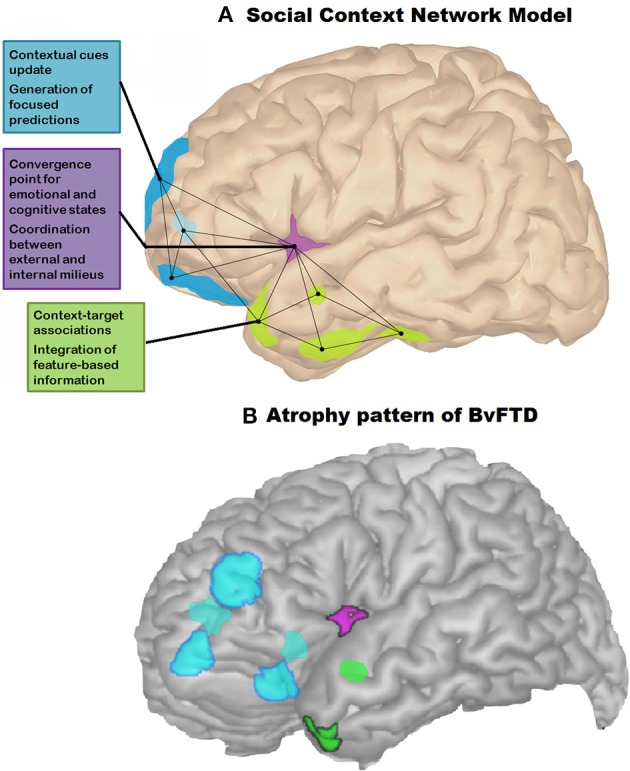
**The social context network model (SCNM). (A)** Lateral view of the left hemisphere showing the fronto-insular-temporal network (light blue, violet, and green regions of interest, respectively). In this network, prefrontal areas would be involved in the generation of focused predictions by updating associations among representations in a specific context. The insular cortex would subserve the convergence of emotional and cognitive states related to the coordination between external and internal milieus. Finally, target-context associations stored in the temporal regions would be integrated with feature-based information processed in frontal regions. Connected nodes represent fronto-insular-temporal interactions. **(B)** Lateral view of earliest regions (frontal-insular-temporal areas) affected in bvFTD. Note the partial overlap with the nodes of the SCNM. Reprinted with permission from Ibañez and Manes (2012).

This focused review showed how context processing plays a relevant role on social cognition impairments in adults with AS. The results of our study suggest that the pattern of social cognition performance of AS individuals may be explained by a single underlying factor. According to a recent social-context network model (SCNM) (Ibañez and Manes, 2012), this factor seems to be the implicit encoding and the integration of contextual information in order to access to social meaning. The SCNM (Figure 3A) proposes that contextual influence on social cognitive processing depends on a fronto-insular-temporal network which: (1) updates contextual cues and uses them to make predictions (frontal areas); (2) coordinates the internal (body) and external (insula) milieus; and (3) consolidates context-social target associative learning (temporal regions). Initially, this model was proposed as an approach to understand social cognition impairments in the behavioral variant of frontotemporal dementia (bvFTD), a neuropsychiatric disease characterized by a progressive deterioration of personality, social behavior, and cognition (Rascovsky et al., 2011). The typical atrophy pattern of bvFTD patients (Figure 3B) involves frontal (e.g., orbitofrontal and ventromedial cortices) and temporal (e.g., amygdala and temporal poles) areas, as well as the insula and white matter tracts between these structures (Rosen et al., 2002; Seeley et al., 2009). Thus, according to the SCNM, the pattern of social cognition deficits in bvFTD reflects a general social-context processing impairment resulting from an abnormal fronto-insular-temporal network.

Although bvFTD and AS have a different onset, course, and clinical presentation, there are important similarities between them. Both are disorders characterized by social dysfunctions and neuropsychiatric symptoms (Midorikawa and Kawamura, 2012). Indeed, AS has been proposed as a differential diagnosis of the non-progressive type of bvFTD (phenocopies) (Hornberger et al., 2009; Midorikawa and Kawamura, 2012). Furthermore, neuroimaging studies have shown that individuals with AS present structural and functional abnormalities in several brain structures, including the cerebellum, the cingulate gyrus, the temporo-parietal junction, and the precuneus (Catani et al., 2008; Lombardo et al., 2011; Via et al., 2011), as well as frontal, temporal, and insular areas (Schultz et al., 2000; Kwon et al., 2004; Welchew et al., 2005; Kosaka et al., 2010). Thus, the contextual social cognition impairments observed in AS may also be partially explained by the abnormal functioning of the fronto-insular-temporal network proposed in the SCNM.

This interpretation may be extended to other neuropsychiatric disorders involving deficits in social cognition domains. For instance, a recent study (Baez et al., 2013b) assessed the performance of patients with schizophrenia and bipolar disorder in social cognition tasks including different levels of contextual dependency and real-life involvement. Similar to adults with AS, both patient groups exhibited deficits in social cognition tasks with greater context sensitivity and real-life involvement. Moreover, temporal and frontal areas are significantly affected in individuals with schizophrenia (Wong and Van Tol, 2003; Amoruso et al., 2012) and, to a lesser degree, in bipolar patients (Harrison, 1999, 2002; Bearden et al., 2001; Frangou et al., 2006). Such patterns indicate that the social cognition deficits present in several neuropsychiatric disorders may be partially explained by a general social-context processing impairment produced by a fronto-insular-temporal network atrophy. The findings described in the present review provide confirmatory evidence for this hypothesis; however, future research should empirically test the assumptions of the SCMN.

Future studies in AS and other neuropsychiatric populations should strictly control for context-dependence levels in social cognition tasks, including measures with context-processing requirements, context-free tests, and experimental manipulations of contextual cues. Moreover, subsequent social cognition studies should consider the importance of studying behavior in truly interactive contexts (Schilbach et al., 2013). Recent methodological advances (Schilbach et al., 2012; Tanabe et al., 2012; Redcay et al., 2013) favor increased ecological validity through the study of social cognition processes (such as gaze) in real-time. These novel paradigms have been successfully employed in participants with ASDs and should also prove useful to investigate other neuropsychiatric disorders. Finally, future studies should establish the specific neural regions and networks involved in social-context processing using ecologically valid paradigms that look at how people actively engage and interact with one another in social encounters (Pfeiffer et al., 2013; Schilbach et al., 2013).

### Conflict of interest statement

Dr. Agustin Ibanez reports having received research funding from CONICYT/FONDECYT Regular (1130920 and 1140114), PICT 2012-0412, and PICT 2012-1309. The author declares that the research was conducted in the absence of any commercial or financial relationships that could be construed as a potential conflict of interest.
